# A combat with the YAP/TAZ-TEAD oncoproteins for cancer therapy

**DOI:** 10.7150/thno.40889

**Published:** 2020-02-18

**Authors:** Ajaybabu V. Pobbati, Wanjin Hong

**Affiliations:** Institute of Molecular and Cell Biology, 61 Biopolis Drive, 138673, Singapore

**Keywords:** TEAD, YAP, TAZ, Hippo, cancer therapy

## Abstract

The transcriptional co-regulators YAP and TAZ pair primarily with the TEAD family of transcription factors to elicit a gene expression signature that plays a prominent role in cancer development, progression and metastasis. YAP and TAZ endow cells with various oncogenic traits such that they sustain proliferation, inhibit apoptosis, maintain stemness, respond to mechanical stimuli, engineer metabolism, promote angiogenesis, suppress immune response and develop resistance to therapies. Therefore, inhibiting YAP/TAZ- TEAD is an attractive and viable option for novel cancer therapy. It is exciting to know that many drugs already in the clinic restrict YAP/TAZ activities and several novel YAP/TAZ inhibitors are currently under development. We have classified YAP/TAZ-inhibiting drugs into three groups. Group I drugs act on the upstream regulators that are stimulators of YAP/TAZ activities. Many of the Group I drugs have the potential to be repurposed as YAP/TAZ indirect inhibitors to treat various solid cancers. Group II modalities act directly on YAP/TAZ or TEADs and disrupt their interaction; targeting TEADs has emerged as a novel option to inhibit YAP/TAZ, as TEADs are major mediators of their oncogenic programs. TEADs can also be leveraged on using small molecules to activate YAP/TAZ-dependent gene expression for use in regenerative medicine. Group III drugs focus on targeting one of the oncogenic downstream YAP/TAZ transcriptional target genes. With the right strategy and impetus, it is not far-fetched to expect a repurposed group I drug or a novel group II drug to combat YAP and TAZ in cancers in the near future.

## Brief history of YAP/TAZ - mediated oncogenesis

**Figure 1:**
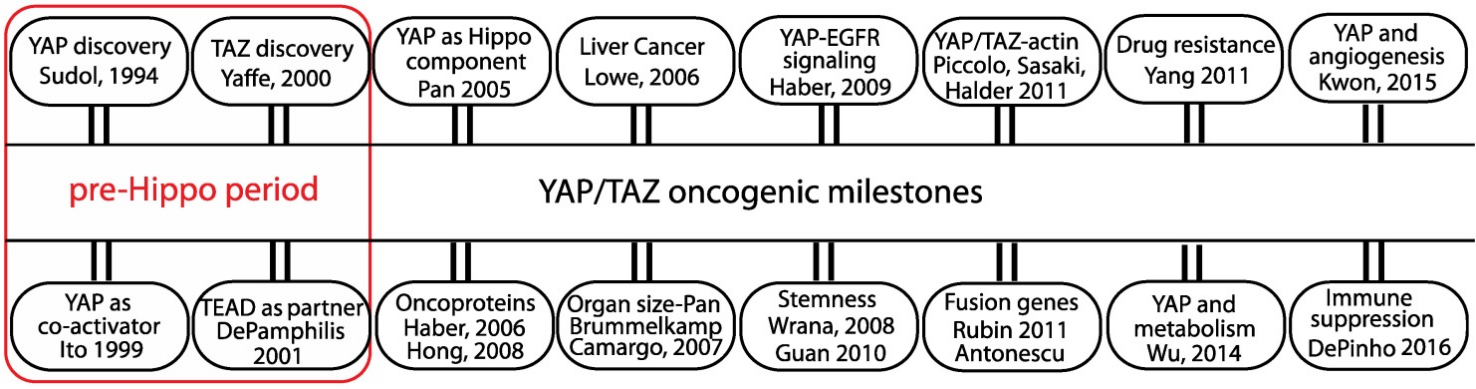
** The oncogenic milestones of the transcriptional co-regulators YAP and TAZ.** Discovery of YAP/TAZ and TEAD functions predate the discovery of the Hippo pathway. Role of YAP/TAZ in the Hippo pathway and the discovery of their oncogenic abilities in cell and animal models are considered significant. The initial studies from the groups that linked YAP/TAZ to oncogenic signaling pathway, stemness, actin cytoskeleton, fusion genes, drug resistance, metabolism, angiogenesis and immune suppression are also listed.

The transcriptional co-regulators YAP (Yes-associated protein) and TAZ (transcriptional co-activator with PDZ-binding motif) are key players that mediate various oncogenic processes and targeting their activities has emerged as an attractive option for potential cancer therapy. YAP, as the name suggests, was initially identified as a protein that associates with Yes, a src family kinase (SFK) 1. The exact function of YAP remained elusive until it was demonstrated to be a potent transcriptional activator 2. YAP's paralog TAZ, identified from a screen for 14-3-3 interacting proteins, is also a transcriptional co-activator 3 (Figure 1).

YAP and TAZ do not have a DNA-binding domain and they need to associate with a transcription factor in order to access DNA. It has now emerged that YAP/TAZ use predominantly the TEAD (TEA domain) family of transcription factors 4 to elicit most of their biologically relevant gene expression programs. ChIP-Seq data unraveled a significant overlap in YAP/TAZ and TEAD peaks throughout the genome, and also showed that some YAP/TAZ-responsive genes are also synergistically regulated by AP-1 transcription factors 5, 6. In addition to its interaction with TEADs, YAP/TAZ also communicates with the mediator complex and chromatin modeling enzymes like the methyltransferase and SWI/SNF complex to elicit changes in gene expression 7-9. YAP/TAZ also suppress gene expression and should be regarded as co-regulators rather than co-activators 10.

YAP/TAZ are now considered as effectors of a physiologically and pathologically important signaling pathway - popularly called the Hippo pathway 11. The Hippo pathway was initially identified in a genetic mosaic screen in *Drosophila* but the pathway components are evolutionarily conserved. It is now known that the primary function of the Hippo pathway is to suppress the activity of Yorkie - the *Drosophila* homolog of YAP 12. The Hippo pathway in mammals also inhibits YAP/TAZ through phosphorylation by the large tumor suppressor (LATS) family of Hippo core kinases 13, which leads to cytoplasmic sequestration via interaction with 14-3-3 proteins and/or degradation via ubiquitin proteasome pathway 14, 15.

YAP and TAZ were first shown to transform mammary epithelial cells 16, 17. The oncogenic role of YAP became apparent when it was shown to be a driver gene in a mouse model of liver cancer 18 (Figure 1). In a conditional transgenic mouse model, YAP overexpression dramatically increases liver size and the mouse eventually develops hepatocellular carcinoma 19, 20. In addition to causing primary tumor growth, YAP also helps in the metastatic dissemination of tumor cells 21.

Over a decade of research has revealed that YAP/TAZ integrates the inputs of various oncogenic signaling pathways, such as EGFR, TGFβ, Wnt, PI3K, GPCR and KRAS. Through expression of the ligand AREG, YAP was first shown to communicate with the EGFR pathway 22 (Figure 1). The genes regulated by YAP/TAZ collectively coordinate various oncogenic processes, such as stemness, mechanotransduction, drug resistance, metabolic reprogramming, angiogenesis and immune suppression (Figure 1), many of which are considered to be cancer hallmarks 23.

YAP and TAZ regulate the expression of crucial transcription factors like Sox2, Nanog and Oct4 and are able to maintain pluripotency or stemness in human embryonic stem cells (ESCs) and in induced pluripotent stem (iPS) cells 24, 25 (Figure 1). More specifically, TAZ has been shown to confer self- renewal and tumorigenic capabilities to cancer stem cells 26. Within the microenvironmental landscape of tissues, YAP/TAZ are increasingly recognized as mechanosensors that respond to extrinsic and cell-intrinsic mechanical cues. To this end, mechanical signals related to extracellular matrix (ECM) stiffness, cell morphology and cytoskeletal tension rely on YAP/TAZ for a mechano-activated transcriptional program 27-29. YAP/TAZ target genes, CTGF and CYR61, cause resistance to chemotherapy drugs like Taxol 30 and YAP/TAZ has emerged as a widely used alternate survival pathway that is adopted by drug-resistant cancer cells 31. YAP/TAZ activity is regulated by glucose metabolism and is connected to the activity of the central metabolic sensor AMP-activated protein kinase (AMPK) 32-35. YAP/TAZ reprograms glucose, nucleotide and amino acid metabolism in order to increase the supply of energy and nutrients to fuel cancer cells 36. Through expression of proangiogenic factors like VEGF and angiopoetin-2 37, 38, YAP is able to stimulate blood vessel growth to support tumor angiogenesis 39. YAP is also shown to recruit myeloid-derived suppressor cells in prostate cancers in order to maintain an immune suppressive environment 40. Active YAP also recruits M2 macrophages to evade immune clearance 41.

A TAZ fusion gene (TAZ-CAMTA1) alone, in the absence of any other chromosomal alteration or mutation, is sufficient to drive epithelioid hemangioendothelioma (EHE), a vascular sarcoma 42, 43. Furthermore, comprehensive analysis of human tumors across multiple cancer types from the TCGA database unraveled that YAP and TAZ are frequently amplified in squamous cell cancers in a mutually exclusive manner 44. In human cancers, there is also a good correlation between YAP/TAZ target gene signature and poor prognosis. To date, a proportion of every solid tumor type has been shown to possess aberrant YAP/TAZ activity. Further, many of the upstream Hippo components that negatively regulate YAP/TAZ are found inactivated across many cancer types 45. Thus, all of this paint a clear picture of the prominent role played by YAP and TAZ at the roots of cancers 46, 47.

## YAP/TAZ inhibiting drugs - combat strategies

**Figure 2:**
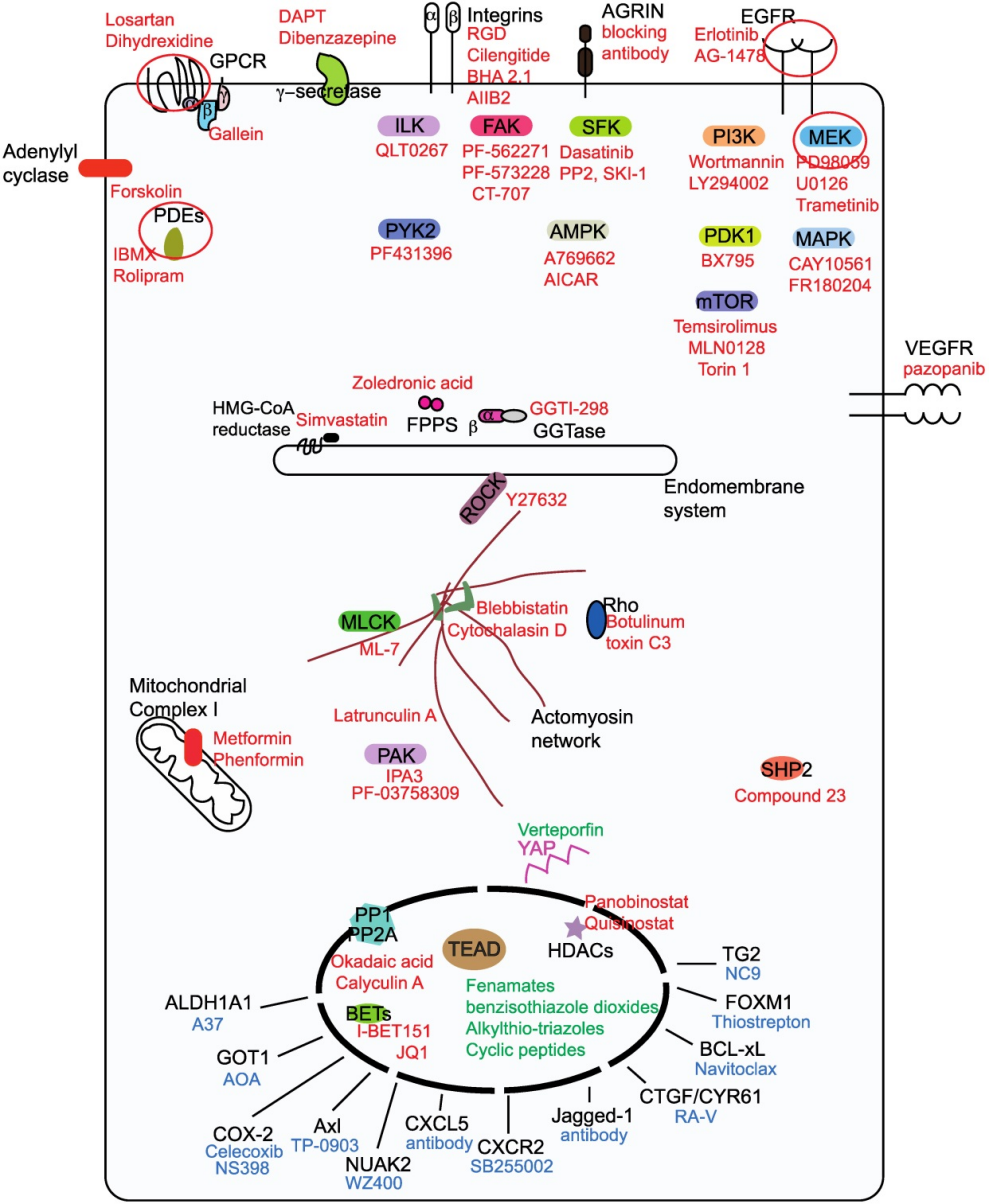
** Classification ofYAP/TAZ-TEAD inhibiting drugs into three groups**. Group I drugs (red font) act upstream and prevent the nuclear entry of YAP and TAZ, group I drug targets for potential pharmacological exploitation in order to generate repurposed YAP/TAZ-inhibiting drugs are circled. Group II drugs (green font) disrupt the formation of the YAP/TAZ-TEAD complex and they primarily bind to the TEAD family of transcription factors. Group Ill drugs (blue font) act on the downstream transcriptional targets in order to prevent YAP/TAZ-mediated oncogenicity.

There are more than fifty drugs that have been shown to inhibit YAP/TAZ activity 48, however, with the exception of verteporfin; none act directly on YAP/TAZ. The unstructured nature of YAP and TAZ renders them difficult to target using small molecules. Therefore, YAP/TAZ inhibition is achieved indirectly through targeting their stimulators or partners. In this review, we focus on small molecules, antibody and peptide-based drugs, as the majority of the drugs in the clinic belong to this class. Less attention is given to nucleotide-based molecules and to small molecule YAP/TAZ inhibitors whose targets are unknown. We classify the YAP/TAZ-inhibiting drugs into three groups with each group having its own combating strategy to counter YAP/TAZ activity (Figure 2). Group I drugs target the upstream YAP/TAZ stimulators and enhance the LATS-dependent inhibitory phosphorylation of YAP/TAZ in order to restrain their transcriptional output. Group II drugs/candidates act directly on YAP/TAZ or TEAD and may either interfere with the formation of the YAP/TAZ-TEAD complex or inhibit TEADs directly and hence affect YAP/TAZ-TEAD transcriptional outcomes. Group III drugs' combat strategy is to target the oncogenic proteins that are transcriptionally upregulated by YAP and TAZ.

## Group I drugs

Group I drugs target the upstream proteins (Figure 2), inhibition of which culminates in the enhancement of the LATS-dependent inhibitory phosphorylation of YAP/TAZ 49, 50. However, some group I targets like SFKs 51-53, AMPK 33, 34 and phosphatases 54-56 act directly on YAP and TAZ and activate them. Majority of group I drugs are kinase inhibitors, in addition to restricting YAP/TAZ nuclear entry; they intriguingly promote TAZ, but not YAP degradation. A possible explanation for this is the presence of two phosphodegrons that render TAZ more prone to degradation 15. Some group I drugs, such as MEK/MAPK inhibitors 57, 58 and γ-secretase inhibitors (GSIs) 59 have the ability to actively reduce both YAP and TAZ levels. HDAC inhibitors however, reduce YAP, but not TAZ levels 60. Here, we have classified the group I drugs based on the nature of the drug target.

### Cell surface receptors

Drugs targeting the EGFR, GPCR, Integrin, VEGFR and adenylyl cyclase families as well as those targeting receptors like the γ-secretase complex and Agrin are shown to inhibit YAP/TAZ activity 51, 61-64.

YAP/TAZ exploits the transformative abilities of the ErbB receptors (EGFR family) to drive cell proliferation. By transcribing ErbB ligands, such as AREG 22, 65, TGF-α 66, NRG1 67 as well as the ErbB receptors EGFR and ErbB3 67, YAP is able to activate ErbB signaling and promote tumorigenesis. Sustained EGFR signaling also disassembles the Hippo core complexes leading to an increased active pool of YAP/TAZ 68 that is ready to transcribe more ErbB ligands/receptors. Under these conditions, EGFR inhibitors like Erlotinib 22 and AG-1478 66 (Figure 2) are able to act as YAP/TAZ inhibitors and may be used for EGFR-driven cancers requiring YAP/TAZ transcription.

Signaling from G-protein coupled receptors (GPCRs), transduced by the associated Gα subunit or by the Gβγ subunits, modulates YAP/TAZ activities 69. Inhibiting Gαq/11 sub-type signaling, using losartan 70, or stimulating Gαs sub-type, using dihydrexidine, has been shown to stimulate YAP inhibitory phosphorylation 69. Agonism of Gαs has been recently exploited to facilitate YAP/TAZ inhibition that reverses fibrosis in mice 71. Gβγ inhibition using gallein has also been shown to restrict YAP/TAZ 72. Activating mutations in the Gαq/11 types of GPCRs present in approximately 80% of the uveal melanoma patients generate an active pool of YAP 73, 74 but the signal transduction occurs via Trio-Rho/Rac signaling and not through the canonical Hippo pathway 74.

Integrin signaling negatively regulates the Hippo pathway complexes to drive YAP/TAZ activity 75, 76. Although blocking integrin activity using RGD peptides 63, cilengitide (cyclic RGD peptide) 77, function-blocking antibodies - BHA 2.1 76 and clone AIIB2 78 has been shown to increase YAP/ TAZ's inhibitory phosphorylation, disappointingly, the efficacy of integrin- blocking drugs against cancers has not been clinically proven 79. Interestingly, a function-blocking antibody against Agrin, an extrinsic stimulator of integrin signaling, abrogates YAP-dependent proliferation in mouse models 63, 80.

Among the kinase inhibitors tested in a biosensor screen for LATS activity, the VEGFR inhibitors are shown to potently activate LATS and thereby inhibit YAP and TAZ activity 81. Further, VEGFR2 signaling is also shown to induce actin cytoskeletal changes and promote YAP/TAZ activation 82. Therefore, VEGFR inhibitors like SU4312, Apatinib, Axitinib and pazopanib are able to inhibit the expression of YAP/TAZ-responsive genes in endothelial cells. But whether these drugs work as YAP/TAZ inhibitors in cancer cells remains to be seen.

Enhancing cyclic AMP (cAMP) levels using the adenylyl cyclase activator forskolin activates the LATS kinases through Protein kinase A (PKA) and Rho 69, therefore forskolin is also a YAP/TAZ inhibitor. cAMP is degraded by the cyclic nucleotide phosphodiesterases (PDE), the use of PDE inhibitors like theophylline, IBMX, ibudilast and rolipram also promotes YAP/TAZ-inhibitory phosphorylation 83, 84.

Notch and YAP/TAZ signaling are also closely linked, inhibiting notch activity by targeting the γ-secretase complex, either using DAPT or dibenzazepine has been shown to decrease YAP/TAZ expression levels in mouse livers and also reduce YAP activation and YAP-induced dysplasia in the intestine 20, 51, 59.

### Intracellular kinases

Integrin signaling activates focal adhesion kinase (FAK), SFK and integrin- linked kinase (ILK). Growth factor and GPCR signaling occurs through mitogen-activated protein kinase (MAPK) and phosphoinositide 3-OH kinase (PI3K) signaling. There is also significant crosstalk in the signaling from these membrane receptors. Given the availability of potent small molecule drugs targeting the downstream kinases, they are leveraged on to inhibit YAP or TAZ activities.

Members of downstream integrin signaling pathway including FAK, its counterpart PYK2, and ILK have emerged as negative regulators of the core Hippo pathway and thus activate YAP/TAZ. Membrane receptors, such as ErbB and GPCRs are unable to activate YAP upon genetic deletion of ILK. Therefore, pharmacological inhibition of ILK using a specific ILK inhibitor, QLT0267 potently inhibits YAP-dependent tumor growth in xenograft models 85. The FAK inhibitors PF-562271 and PF-573228 have also been shown to enhance the LATS-mediated inhibitory phosphorylation of YAP 63, 75. A multi-kinase inhibitor CT-707 that predominantly inhibits FAK, anaplastic lymphoma kinase (ALK) and PYK2 is able to render cancer cells vulnerable to hypoxia through YAP inhibition 86. Inhibiting PYK2 activity using the dual PYK2/FAK inhibitor PF431396 destabilizes TAZ and also inhibits YAP/TAZ activity in triple negative breast cancer cells 87.

The SFK member Src prevents the activation of LATS 75, 88, thereby relieves YAP/TAZ inhibition by LATS. Interestingly, SFKs, Src and YES are also shown to activate YAP through direct tyrosine phosphorylation 51-53. Treating cells with SFK inhibitors, such as Dasatinib, PP2, SU6656, AZD0530 and SKI-1 inactivates YAP 51-53, 75, 88. In β-catenin-driven cancers, YES facilitates the formation of a tripartite complex comprising β-catenin, YAP and TBX5 that drives cell survival and tumor growth 53, 89. The SFK inhibitor dasatinib also serves as YAP inhibitor in these cancers 53. Dasatinib, in addition to inhibiting SFKs may also potently inhibit PDGFR and Ephrin receptors, both of which are known to activate YAP/TAZ 90, 91. However, FAK and SFK inhibitors have shown very limited efficacy against solid tumors in clinical trials therefore their utility in YAP-driven cancers remains to be seen.

MEK (MAP kinase kinase) and YAP interact with each other and maintain transformed phenotypes in liver cancer cells 57. MEK inhibitors PD98059, U0126 and trametinib or MAPK inhibitors CAY10561 and FR180204 are able to trigger degradation of YAP in a Hippo-independent manner 57, 58. The finding that MEK inhibition causes YAP degradation is, however, difficult to reconcile if YAP and TAZ are shown to mediate resistance to the MEK inhibitor trametinib 92. The efficacy of trametinib is also being evaluated in EHE, a cancer that is caused by the TAZ-CAMTA1 fusion gene (NCT03148275).

PI3K inhibitors Wortmannin/LY294002 as well as the drug BX795, an inhibitor of its effector 3'-phosphoinositide-dependent kinase-1 (PDK1) prevents nuclear entry of YAP 68. PI3K is closely linked to the mammalian target of rapamycin (mTOR) pathway. mTOR inhibitors temsirolimus and MLN0128 have been shown to inhibit YAP activity in patients with idiopathic pulmonary fibrosis and in a mouse model of cholangiocarcinoma, respectively 93, 94. YAP levels in TSC1 mutant mouse could also be reduced by blocking mTOR using torin1 treatment that induces the autophagy-lysosomal pathway 95.

YAP/TAZ inhibition is an additional unexpected activity possessed by the few kinase inhibitors mentioned above. However, apart from YAP/TAZ inhibition, all other signaling events initiated by the target kinase are also shut down due to inhibitor treatment. If these signaling events are critical for cellular homeostasis, then, toxic side effects will outweigh clinical benefits and this cannot be uncoupled from YAP/TAZ inhibition. Therefore, kinase inhibitors that failed in the trials due to unacceptable toxcity or poor pharmacokinetics may not be repurposed as YAP/TAZ inhibitors in the clinic. Focus should be on the kinase inhibitors that are already in the clinic like EGFR, VEGFR, MEK, PI3K or mTOR inhibitors but efficacy needs to be proven in order to repurpose them as YAP/TAZ inhibitors. The kinase targeted by the inhibitor must activate YAP/TAZ in tumors, for the treatment to be efficacious and this restricts the use of kinase inhibitors to selective tumor types. Intriguingly, YAP/TAZ activation has emerged as a prominent survival strategy adapted by cancers that cause drug resistance to EGFR and its downstream MEK/MAPK inhibitors 31. In such scenarios, coupling a group II YAP/TAZ inhibitor with a EGFR pathway inhibitor might offer the intended treatment benefits.

### Mevalonate pathway inhibitors

The mevalonate pathway is essential for the biosynthesis of isoprenoids, cholesterol and steroid hormones. Statins as well as other mevalonate pathway inhibitors like zoledronic acid and GGTI-298 that target farnesyl pyrophosphate synthase and geranylgeranyltransferase, respectively are identified as drugs that restrict the nuclear entry of YAP and TAZ 96, 97. Studies have also shown that combining statins like simvastatin with the EGFR inhibitor gefitinib provides stronger anti-neoplastic effects 98. Atorvastatin and zoledronic acid have entered Phase II clinical trials in triple negative breast cancer to test if they improve the pathological complete response rates (NCT03358017).

### Actin modulators

Actin polymerization promotes YAP/TAZ nuclear localization and therefore, polymerization inhibitors like latrunculin A 27 and cytochalasin D 28, 29 inhibit YAP/TAZ. Myosin or myosin light-chain kinase inhibitors like blebbistatin and ML-7, respectively have a similar effect 27, 29. Interfering with the actomyosin cytoskeleton through other means, such as Rho inhibition (toxin C3 treatment), or by using Rho kinase inhibitors like Y27632 has also been shown to have an inhibitory effect on YAP/TAZ 27, 29. p21 activated kinase (PAK) family kinases are cytoskeletal regulators as well as Hippo inhibitors. The PAK allosteric inhibitor IPA3 prevents YAP's nuclear entry 63, 99, further, the PAK4 inhibitor PF-03758309 is also shown to reduce YAP levels 77.

### Phosphatase inhibitors

YAP/TAZ inhibitory phosphorylation is dynamic and the protein phosphatases PP1 and PP2A are shown to associate with YAP/TAZ and aid in their dephosphorylation and activation. Inhibiting these phosphatases using okadaic acid or calyculin A increases YAP/TAZ phosphorylation and shifts YAP/TAZ to the cytoplasm 54-56. Some of the oncogenic functions of YAP/TAZ are also mediated by the protein-tyrosine phosphatase SHP2 100, therefore SHP2 inhibitors have also been shown to attenuate YAP/TAZ activity 101.

### Cellular energy stress modulators

Cellular energy stress is closely linked with attenuation of YAP/TAZ activities 32. Drugs that enhance energy stress like the mitochondrial complex I inhibitors metformin and phenformin, enhance YAP/TAZ inhibitory phosphorylation, cytoplasmic localization and suppression of YAP/TAZ- mediated transcription 32. The energy stress induced by these drugs activates AMPK, which is shown to phosphorylate and stabilize AMOTL1 - a YAP/TAZ negative regulator 32. AMPK is also shown to directly phosphorylate and inactivate YAP by disrupting its interaction with TEADs 33, 34. Therefore, AMPK activators A769662 and AICAR (an AMP-mimetic) are YAP inhibitors 32-34.

### Epigenetic modulators

Histone deacetylases (HDACs) are uniquely positioned to alter the transcription of target genes. Interestingly, HDAC inhibitors panobinostat, quisinostat, dacinostat, vorinostat and Trichostatin A transcriptionally repress the expression of YAP but not TAZ, and thereby reduce YAP-addicted tumorigenicity 60. Treatment of cholangiocarinoma cells with the HDAC inhibitor CG200745 is also shown to decrease YAP levels 102. Although HDAC inhibitors are used to treat hematological malignancies their efficacy in solid cancers is questionable, however, combining HDAC inhibitor panobinostat with BET (bromodomain and extra-terminal) inhibitor I-BET151 achieves more effective YAP inhibition 103. There is also a clinical trial designed to evaluate the efficacy of HDAC/BET inhibitor combination in solid tumors and determination of YAP expression level after drug treatment is used as one of the objectives (NCT03925428). The BET family protein BRD4 is a part of the YAP/TAZ-TEAD transcriptional complex and inhibiting BRD4 using BET inhibitor JQ1 inhibits YAP upregulation and YAP-mediated transcription in KRAS mutant cells 104.

Many group I drugs can potentially be repurposed to treat YAP/TAZ- driven cancers 105. Among the group I drugs, only statins, trametinib and HDAC/BET inhibitors are being evaluated in clinical trials to test if they act against YAP/TAZ. Our prediction is that group I drugs that facilitate YAP/ TAZ inhibitory phosphorylation as well as degradation will have greater success in combating YAP/TAZ in cancers as YAP/TAZ degradation prevents their reactivation through other mechanisms. Importantly, the repurposing of group I drugs would also allow YAP/TAZ and its target gene(s) expression-based stratification amongst cancer patients.

## Group II modalities

**Figure 3:**
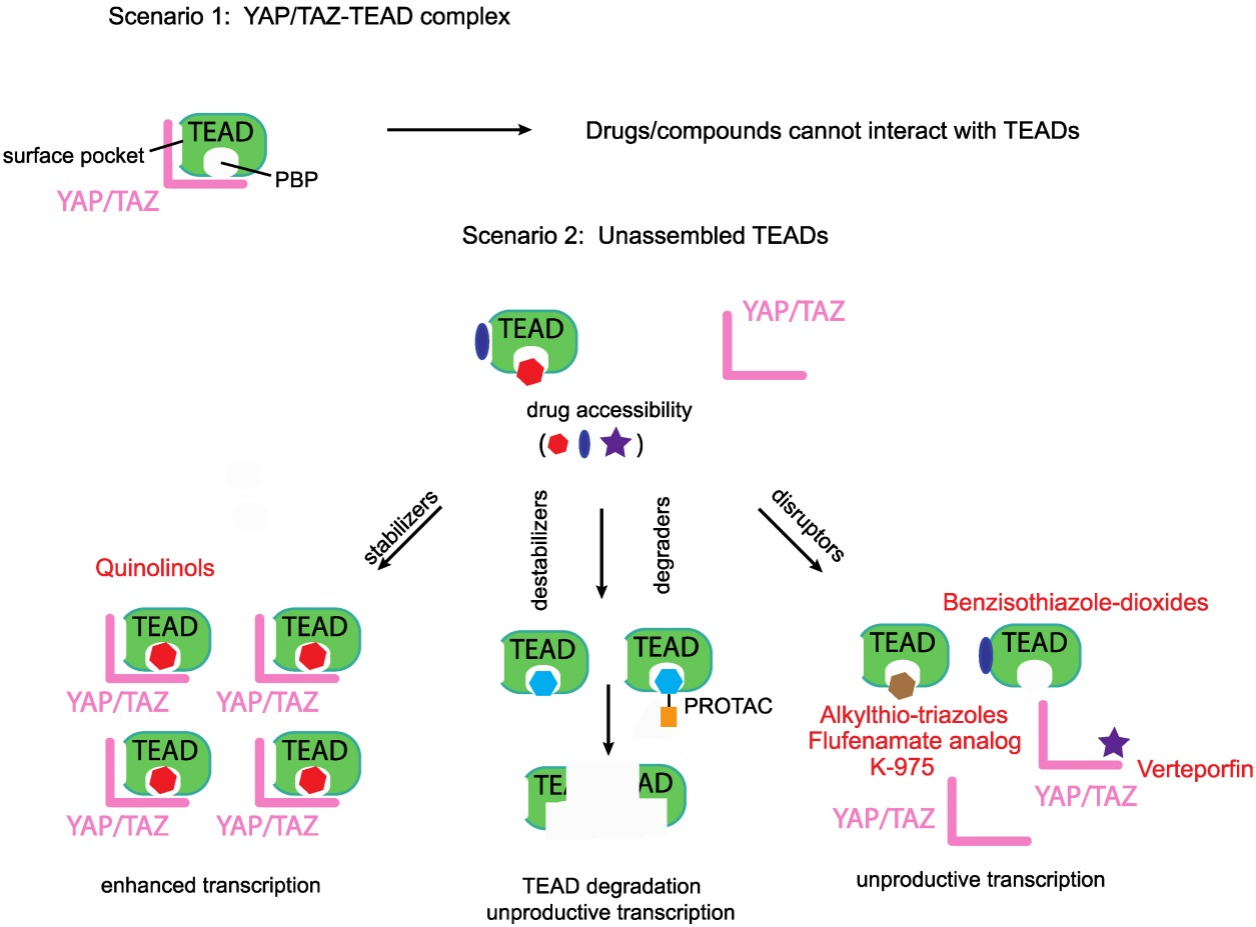
** Disruptors, stabilizers and destabilizers/degraders.** A preformed YAP/TAZ-TEAD complex prevents access to drugs that occupy either the TEADs' surface or the palmitate-binding pocket (PBP), however, unassembled TEADs are accessible to drugs. Majority of the known YAP/TEAD-binding compounds are disruptors as they prevent the formation of the YAP/TAZ-TEAD complex. Two other classes of TEAD-binding compounds are stabilizers and destabilizers/degraders. Stabilizers either stabilize TEAD expression levels or enhance the formation of the YAP/TAZ-TEAD complex. Destabilizers bind to TEADs' surface or PBP and reduce TEAD expression levels through *in situ* denaturation, degraders on the other hand direct TEADs for proteasomal degradation.

Modalities that target either the TEAD family of transcription factors or YAP/TAZ are classified under this group (Figure 3). The majority of the modalities, with the exception of verteporfin 106, target TEADs and are therefore predicted to act in the nucleus. By pairing with the TEAD family of transcription factors, YAP and TAZ upregulate the expression of many oncoproteins. The C-terminus of all TEADs possesses the YAP/TAZ-binding domain. The partnership between YAP/TAZ and TEAD is essential for the initiation of transcriptional program to drive oncogenesis. YAP is no longer oncogenic when sequestered by a dominant negative TEAD that lacks the DNA-binding domain 106. Similarly, a naturally occurring DNA-binding deficient TEAD isoform is also able to inhibit YAP/TAZ-mediated oncogenicity 107. Therefore, directly inhibiting TEAD or preventing YAP/TAZ-TEAD interaction is a promising and most direct strategy that warrants special attention 108.

Group I drugs target the upstream YAP/TAZ-activating proteins like the EGFR, GPCR, Src, or Integrins. As there are so many upstream YAP/TAZ activators, group I drugs are vulnerable to oncogenic bypass where inhibition of one group I YAP/TAZ activator leads to selection of cancer cells that activate YAP/TAZ via another group I activator. Strategically, Group II drugs may address this issue by directly targeting YAP/TAZ or TEAD, the converging points for various pathways and also the effectors for oncogenic transcription. However, Group II targeting modalities are still at the exploratory stage and it remains to be seen whether it is feasible to develop a Group II modality that works in clinic. We also need to be mindful of the possible associated toxicities due to YAP/TAZ-TEAD inhibition 109.

Most of the reported Group II modalities are disruptors; they target YAP/TAZ or TEAD and prevent their binary interaction. However, in addition to disruptors, in the future, we predict the emergence two other classes of group II compounds that would act as TEAD stabilizers and destabilizers/degraders (Figure 3).

### Disruptors targeting YAP

A small molecule benzoporphyrin drug named Verteporfin (VP) was shown to have the ability to bind to YAP and disrupt the YAP-TEAD interaction 106. VP is also able to inhibit YAP-induced excessive cell proliferation in YAP- inducible transgenic mice and in NF2 (upstream Hippo pathway component) liver-specific knockout mouse models 106. Although we do not understand the molecular details of VP binding to YAP, it is still undoubtedly the most popular YAP inhibitor within the scientific community. However, we need to be cautious as some of the tumor-inhibitory effects of VP are reported to be YAP- independent 110, 111. VP is photosensitive and proteotoxic and there is a need for better derivatives. A VP derivative, a symmetric divinyldipyrrine was shown to inhibit YAP/TAZ-dependent transcription but it is not clear if the compound specifically binds to YAP 112.

### Disruptors targeting the TEADs' surface

YAP and TAZ bind on the TEADs' surface; Inventiva Pharma has identified several compounds with benzisothiazole-dioxide scaffold that bind to the TEADs' surface and disrupt the YAP/TAZ-TEAD interaction. These compounds are currently in the lead optimization stage and have the potential to treat malignant pleural mesothelioma as well as lung and breast cancers that are driven by YAP/TAZ 113.

YAP cyclic peptide (peptide 17) and cystine-dense peptide (TB1G1) are also disruptors of YAP/TAZ-TEAD interaction *in vitro* but they have poor cell-penetrating abilities 114, 115. Interestingly, a peptide derived from the co-regulator Vgll4 appears to have remarkable cell-penetrating abilities and inhibits YAP-mediated tumorigenesis in animal models 116. Vgll proteins, named Vgll1-4 in mammals, belong to another class of co-regulators that pair with TEADs in a structurally similar, and therefore, in a mutually exclusive manner with YAP and TAZ 117, 118.

### Disruptors targeting TEADs' palmitate-binding pocket (PBP)

We identified a novel druggable pocket in the center of the TEADs' YAP/TAZ- binding domain 119 that could be occupied by fenamate drugs. Palmitate was subsequently shown to occupy this pocket, hereafter referred to as the palmitate-binding pocket (PBP). TEAD palmitoylation is shown to be important for stability and for the interaction with YAP 120, 121. Although the fenamate drug flufenamic acid competes with palmitate for binding to TEAD, higher concentrations are needed for it to be effective and it is not a disruptor of the interaction between YAP/TAZ with TEADs 122. However, covalently linking the fenamate to TEAD, using a chloromethyl ketone substitution, enables it to disrupt the YAP-TEAD interaction 123. The non-fused tricyclic compounds identified by Vivace Therapeutics could also be considered as fenamate analogs but it remains to be seen if they function as disruptors 124. Through structure-based virtual screen, vinylsulfonamide derivatives were identified as compounds that bind to PBP 125. Optimization of these derivatives yielded DC-TEADin02 a covalent TEAD autopalmitoylation inhibitor with an IC50 value of 200 nM. Interestingly, DC-TEADin02 is able to inhibit TEAD activity without disrupting the YAP-TEAD interaction.

Palmitate, by occupying the PBP, allosterically modulates YAP's interaction with TEAD 121, therefore it is conceivable that there might be small molecules that occupy the PBP and allosterically disrupt YAP/TAZ's interaction with TEADs. To this end, Xu Wu's group has identified and patented several potent compounds with alkylthio-triazole scaffold as PBP- occupying compounds that prevent YAP-TEAD interaction in cells 126. Another potent TEAD inhibitor that occupies the PBP is the small molecule K-975 127. K-975 also disrupts the YAP-TAZ-TEAD interaction and displayed anti-tumorigenic properties in malignant pleural mesothelioma cell lines much akin to the loss of YAP. Although palmitate is covalently attached to TEAD, it is a reversible modification and addition of PBP-occupying small molecules reduce the cellular palmitoylation status of TEADs 126. Moreover, the palmitoyl group is also removed from TEADs by depalmitoylases 128.

Being predominantly unstructured, YAP and TAZ are difficult to target directly. However, TEADs offer two attractive ways for targeting, one is to directly block the YAP/TAZ-binding pocket on the TEADs' surface with small molecules or peptides, whilst the other is to leverage on the PBP and allosterically disrupt YAP/TAZ interaction or inhibit TEADs (Figure 3). However, the molecular determinants that confer YAP/TAZ disrupting ability to PBP-occupying small molecules are not clear. We do not know why flufenamate and DC-TEADin02 are unable to disrupt YAP/TAZ-TEAD interaction, like chloromethyl fenamate, K-975 and compounds with alkylthio-triazole scaffold.

### Stabilizers and destabilizers/degraders

The PBP could also be leveraged to allosterically enhance YAP/TAZ-TEAD stability or interaction. This prediction is subject to the identification of small molecules that functionally mimic the ligand palmitate (Figure 3). Compounds with such an ability will enhance TEAD-dependent transcription and may have therapeutic value for regenerative medicine where enhancement of YAP/TAZ- TEAD activity is needed to repair damaged tissues 129. We recently identified that quinolinols occupy the PBP, stabilize YAP/TAZ levels and upregulate TEAD-dependent transcription 130. Enhanced YAP/TAZ levels increase the pool of assembled YAP/TAZ complex and therefore quinolinols could be considered as stabilizers (Figure 3).

We identified a few chemical scaffolds that have the ability to occupy the PBP and destabilize TEAD (unpublished results). Addition of these compounds unfolds the TEADs' YAP/TAZ-binding domain and we call these compounds destabilizers (Figure 3). Degraders could be generated when potent and selective TEAD surface or PBP-occupying compounds are coupled to proteolysis targeting chimera (PROTAC) 131 to direct TEAD proteasomal degradation. Therefore, destabilizers aim to reduce the cellular concentration of TEADs through *in situ* unfolding and degraders reduce TEAD levels through proteasomal degradation. Reducing the levels of their interacting partners deprives YAP/TAZ of their ability to activate transcription.

Any TEAD-binding compounds (disruptors, stabilizers or destabilizers/degraders) can only access unbound TEADs, as binding of YAP and TAZ blocks both the surface and the palmitate-binding pockets (Figure 3). After accessing unbound TEADs, the disruptors and destabilizers/degraders reduce, whereas the stabilizers enhance, the formation of the YAP/TAZ-TEAD complex.

## Group III drugs

YAP/TAZ-mediated tumor development is due to the collective action of the repertoire of proteins that are expressed under their influence. However, some proteins are able to drive oncogenesis much better than others and they vary depending on the solid tumor and context. Therefore, drugs against these downstream YAP/TAZ targets including metabolic enzymes, kinases, ligands and proteins, such as BCL-xL, FOXM1 and TG2 are also used to combat YAP/TAZ-mediated oncogenicity (Figure 2).

### Metabolic enzymes

TAZ-dependent expression of ALDH1A1 (aldehyde dehydrogenase) is shown to impart stemness and tumorigenic ability; inhibition of ALDH1A1 using A37 reverses this transformation 132. GOT1 - the aspartate transaminase induced by YAP/TAZ, confers glutamine dependency to breast cancer cells and targeting this metabolic vulnerability using aminooxyacetate (AOA) represses breast cancer cell proliferation 133. Targeting the YAP/TAZ transcriptional target cyclooxygenase 2 (COX-2) using celecoxib inhibits cell proliferation and tumorigenesis in NF2 mutant cells 134. Interestingly, a positive feedback is seen in hepatocellular carcinoma cell lines where COX-2 is also shown to increase the expression of YAP 135. Inhibiting COX-2 using NS398 stimulates LATS-dependent phosphorylation of TAZ 136.

### Kinases

In hepatocellular carcinoma, Axl kinase has been shown to be crucial for mediating several YAP-driven oncogenic functions like cell proliferation and invasion 137. Similarly, YAP-driven Axl expression has been implicated in the development of resistance against EGFR inhibitors in lung cancer and sensitivity could be restored through Axl inhibition using TP-0903 138. YAP is shown to upregulate the expression of the kinase NUAK2 139 that, in turn activates YAP/TAZ by inhibiting LATS. Specific pharmacological inhibition of NUAK2 using WZ400 shifts YAP/TAZ to the cytoplasm and reduces cancer cell proliferation 140.

### Ligands

In a mouse model of prostate adenocarcinoma, the YAP-TEAD complex promotes the expression of the chemokine ligand CXCL5 that facilitates myeloid-derived suppressor cells (MDSC) infiltration and adenocarcinoma progression. Administering CXCL5 neutralizing antibody, or blocking CXCL5 receptor using the inhibitor SB255002, inhibits MDSC migration and tumor burden 40. The notch ligand Jagged-1 that is upregulated by YAP/TAZ is crucial for liver tumorigenesis 59, 141. Treating liver tumor cells with Jagged-1 neutralizing antibody greatly reduces oncogenic traits. The levels of integrin ligands CTGF and CYR61 that are also YAP target genes, could be reduced using the cyclopeptide RA-V (deoxybouvardin) leading to a reduction of YAP- mediated tumorigenesis in mst1/2 (Hippo homolog) knockout mouse model 142. Although neutralizing CTGF (FG-3019/pamrevlumab) and CYR61 (093G9) antibodies are available, they have not been effectively used against YAP/TAZ-driven cancers.

### BCL-xL

YAP mediates drug resistance to RAF- and MEK-targeted therapies in BRAF V600E cells, in part through the expression of the anti-apoptotic protein BCL- xL. BCL-xL inhibition using navitoclax sensitizes these cells to targeted therapies 92.

### FOXM1

YAP-mediated proliferation through its target gene FOXM1 could be prevented in sarcoma cell lines and mouse models through the administration of thiostrepton that reduces FOXM1 levels 143.

### Transglutaminase 2

Transglutaminase 2 (TG2) - the multifunctional transamidase is a YAP/TAZ target gene that is important for cancer stem cell survival and for maintaining integrin expression. TG2 inhibition using NC9 dramatically reduces tumorigenicity 144, 145.

We are aware that many of these target proteins also act upstream and stimulate YAP/TAZ by forming a positive feedback but we would nevertheless consider them in this group and not as group I as their expression is influenced by the TEAD-binding motif and YAP/TAZ.

## Major challenges

Although attractive, toxicity issues and the identification of responsive patient population could be challenges in the successful implementation of the YAP/TAZ inhibitors in the clinic. YAP/TAZ inhibition might elicit toxicity 146; homozygous disruption of YAP in mice causes embryonic lethality, whereas TAZ knockouts are viable 147-150. Tissue-specific deletions of YAP in the heart 151, lung 152 or kidney 153 cause hypoplasia, whereas YAP/TAZ deletion in the liver cause hepatomegaly and liver injury 154. Surprisingly, YAP/TAZ knockouts in the intestine are well tolerated with no apparent tissue defects 155. All of these suggest that YAP and TAZ are crucial for development. However, they appear to be dispensable for adult tissue homeostasis. In most adult tissues, under normal homeostasis, YAP/TAZ are found restricted to the cytoplasm and are activated primarily in response to injury to initiate tissue regeneration. Therefore, it is predictable that administration of a YAP/TAZ inhibitor may not elicit severe toxicity. However, given the dynamic shuttling of YAP/TAZ/Yorkie between nucleus and cytoplasm 156-158, it is feasible that they still have a role in normal tissue homeostasis. Fittingly, YAP has been identified to be important for podocyte homeostasis and its functional inactivation compromises the glomerular filtration barrier and cause renal disease 109. Along similar lines, renal toxicity was observed in mice administered with K-975 - a YAP/TAZ-TEAD inhibitor 127. Renal toxicity in targeted therapy is very common and is seen in most of the kinase inhibitors used in oncology 159. Yet these kinase inhibitors are in the clinic as there is a therapeutic window, where the drug could be dosed to improve patient survival without causing much toxicity. The same could be envisaged for YAP/TAZ-inhibiting drugs.

Several drugs that act as YAP/TAZ inhibitors target multiple signaling pathways. Targeting multiple pathways could be a boon or a bane. Drug resistance is minimized in a multi-targeted approach as potential bypass mechanisms are also targeted. However, toxicity becomes an issue when the drug targets multiple important signaling pathways. For instance, raising cAMP through the use of PDE inhibitors activates a multitude of proteins like PKA, EPACs, ion channels and small GTPases. Similarly, GPCR modulators influence multiple pathways through signaling via G proteins, arrestins or GPCR kinases. To reduce toxic side effects, there are options available like selective targeting or biased signaling. Instead of hitting all the PDEs, the PDE enzyme that is the most potent activator of YAP/TAZ should be selectively targeted. Nonspecific PDE inhibitors cause more severe side effects than sub-type selective PDE inhibitors 160. Similarly, through stabilizing a particular GPCR conformation, certain small molecule GPCR modulators are able to effect signaling bias where one GPCR effector is preferentially activated over others, say G proteins over β-arrestins, this way only a subset of signaling pathways get activated 161.

Another major challenge is the identification of patients responding to a YAP/TAZ inhibitor. YAP/TAZ expression is low in normal tissues and their levels are significantly elevated in cancers. Is YAP or TAZ positivity in tumors sufficient criteria to administer a YAP/TAZ inhibitor? YAP and TAZ might not be transcriptionally active or drivers in all tumors. Further, they could be expressing target genes that negatively regulate their activity 162, 163. There are also tumor types where YAP/TAZ or TEAD levels have no prognostic significance 46. These YAP/TAZ positive tumors are unlikely to respond to a YAP/TAZ inhibitor. Barring a few such scenarios, in many solid tumors, YAP or TAZ expression levels correlate well with higher-grade cancers or poor prognosis. Tumors with nuclear YAP or TAZ that are also positive for the downstream oncogenic YAP/TAZ target genes are likely to respond to a YAP/TAZ inhibitor and this should be used as criteria for patient stratification. As many of the YAP/TAZ-TEAD target genes are secreted proteins, the expression levels of these in the serum could also be estimated in addition to assessing their levels through immunohistochemistry.

## Conclusions and future perspectives

As YAP and TAZ contribute to the acquisition of many hallmarks of cancer traits, targeting them is predicted to be more relevant for the management of several cancer types. It is still early to expect a newly developed drug against YAP/TAZ but it is nevertheless disconcerting to see that there are hardly any clinical trials that evaluate if known drugs could be repurposed as YAP/TAZ- inhibitors. Group I drugs are well suited to repurpose 105 but only statins (NCT03358017); trametinib (NCT03148275) and epigenetic modulators (NCT03925428) are being evaluated in clinical trials, assessment of the expression levels of YAP/TAZ after drug treatment is used as one of the clinical trial objectives. It is essential that we bolster our pharmacological arsenal so that we are prepared to combat YAP and TAZ. Group I drugs that failed in oncology trials are not expected to fare any better against YAP/TAZ. However, drugs that are already in the clinic like the kinase inhibitors targeting the EGFR or MEK, PDE inhibitors as well as GPCR modulators could be repurposed to combat YAP/TAZ. The cancer types need to be carefully stratified to ensure they are driven by YAP/TAZ through the upstream stimulator targeted by the drug. To overcome potential bypass mechanism or drug resistance, combinatory use of group I and II drugs could also serve as an avenue for cancer treatment. For the group III drugs, the situation may not be as promising, as they target only one of the many possible oncogenic proteins regulated by YAP/TAZ. Again, combinatory inhibition of few downstream target genes could be considered if they are collectively essential for oncogenic manifestation of YAP/TAZ-driven transcription. As they are new and untested, there is much excitement and progress in the development of novel group II compounds as drugs against YAP/TAZ. We are at an exciting juncture in the Hippo field where we could potentially see a novel group II drug or a repurposed group I drug to combat YAP/TAZ in the near future.
